# Comparison of cognitive workload and surgical outcomes between a three-dimensional and conventional microscope macular hole surgery

**DOI:** 10.1186/s12886-024-03361-5

**Published:** 2024-03-01

**Authors:** Aditya Kelkar, S. Natarajan, Akshay Kothari, Mounika Bolisetty

**Affiliations:** 1 National Institute Of Ophthalmology, 1187/30, off Ghole road, near Phule Museum, 411005 Pune, Maharashtra India; 2 Aditya Jyot eye hospital, Mumbai, India

**Keywords:** Macular Hole, Conventional microscope, 3D viewing system, Cognitive workload

## Abstract

**Background:**

Performing a surgical task subjects the surgeon to multitudinal stressors, especially with the newer 3D technology. The quantum of cognitive workload using this modern surgical system in comparison to the Conventional microscope system remains unexplored. We evaluate the surgeon’s cognitive workload and the surgical outcomes of macular hole(MH) surgery performed on a 3D versus a Conventional microscope operating system.

**Methods:**

50 eyes of 50 patients with MH undergoing surgery using the 3D or Conventional microscope visualization system. Cognitive workload assessment was done by real-time tools(Surgeons’ heart rate [HR] and oxygen saturation[SPO2]) and self-report tool(Surgery Task Load Index[SURG-TLX] questionnaire) of three Vitreoretinal surgeons. Based on the SURG-TLX questionnaire, an assessment of the workload was performed.

**Results:**

Of the 50 eyes, 30 eyes and 20 eyes underwent surgery with the Conventional microscope and the 3D system, respectively. No difference was noted in the MH basal-diameter(*p* = 0.128), total surgical-duration(*p* = 0.299), internal-limiting membrane(ILM) peel time(*p* = 0.682), and the final visual acuity (VA; *p* = 0.515) between the two groups. Both groups showed significant improvement in VA(*p* < 0.001) with a 90% closure rate at one-month post-surgery. Cognitive workload comparison, the intraoperative HR(*p* = 0.024), total workload score(*P* = 0.005), and temporal-demand dimension(*p* = 0.004) were significantly more in Conventional microscope group as compared to 3D group. In both the groups, the HR increased significantly from the baseline while performing ILM peeling and at the end.

**Conclusion:**

The surgeon’s cognitive workload is markedly reduced while performing macular hole surgery with a 3D viewing system. Moreover, duration of surgery including ILM peel time, MH closure rates, and visual outcomes remains unaffected irrespective of the operating microscope system.

**Supplementary Information:**

The online version contains supplementary material available at 10.1186/s12886-024-03361-5.

## Background

There have been substantial developments in the surgical procedures and instrumentation of vitreoretinal surgery in the five decades since Robert Machemer performed the first pars plana vitrectomy [1]. The technology of surgical microscopes and operating procedures, however, has essentially remained unaltered. When compared to other ocular surgical procedures, VR surgery is both more complicated and time-consuming. Working for an extended period using conventional microscope systems can be harmful to the musculoskeletal system due to their poor ergonomics [2–4]. The detrimental neck and back postures with these conventional microscope operating systems have been observed to reduce surgical longevity significantly [2–4]. The emergence of a three-dimensional (3D) imaging system in ocular surgery is a significant advance since it improves ergonomics, depth of field, patient cooperation due to decreased illumination, lower retinal phototoxicity, and visualization and learning for the surgical team [5–8]. This cutting-edge 3D technology has been progressively employed for a variety of anterior and posterior segment surgeries throughout the course of the previous five years [5–9]. 

The operating room presents the surgeon with a variety of stressful situations when doing surgery [10, 11]. Every surgeon, no matter how skilled or experienced, experiences some degree of cognitive challenge during surgery [10, 11]. It is possible for the cognitive effort to become overwhelming under certain stressful circumstances [10, 11]. This may seriously affect the surgeon’s performance, which may then affect the patient’s safety [10, 11]. The surgeon must make quick adjustments and continually revise the surgical procedure in response to the evolving intraoperative circumstances while performing a surgery. This not only assesses his visual and motor skills, but also his mental preparedness, cognitive agility, and ability to cope with cognitive pressure [12]. An essential performance indicator that can support and improve his cognitive capacities is measuring the cognitive workload. Self-report tools like the National Aeronautics and Space Administration task load index (NASA-TLX), SWAT (Subjective Workload Assessment Technique), and real-time tools like measuring heart rate (HR), eye tracking, and electroencephalography (EEG) have been used to quantify cognitive load [13]. The Surgery Task Load Index (SURG-TLX) was created in 2011 as a modified version of the NASA-TLX [14]. It is a validated, multidimensional, surgery-specific workload measure that can be used to measure the cognitive load of surgeons [14]. 

The 3D surgical system offers several benefits, however, transitioning to a modern operating system can increase a surgeon’s cognitive load. When compared to the traditional microscope system, Romano et al. [15] found that 3D vitrectomy was more comfortable for the surgeon. Their results, however, were not drawn from validated methodology and so were not totally supported [15]. To better understand this attribute, we compared the cognitive workload, surgical time, and visual outcomes of macular hole (MH) surgery using the 3D viewing system to the conventional microscope (CM).

## Methods

It was a prospective, comparative, non-randomized, interventional study carried out at two tertiary eye care hospitals in western India. The study was conducted in accordance to the tenets of the Declaration of Helsinki and was approved by the Institutional Review Board. Written informed consent was obtained from all patients.

All patients with idiopathic MH who presented to the VR services between September 2020 and August 2021 were included in the study. Eyes with any other ocular co-morbidities like diabetic retinopathy, glaucoma, macular degeneration, uveitis, or eyes with a previous history of any other ocular surgery apart from cataract surgery with intra-ocular lens implantation were excluded. The patients were randomly assigned to have surgery with either the CM (Zeiss Lumera 700, Carl Zeiss Meditech, USA; Group 1) or the Artevo 800 3D digital visualization system (Carl Zeiss Meditech, USA; Group 2), based on the surgeon’s preference. All patients underwent the standard surgical procedure of 25G pars plana vitrectomy with an internal limiting membrane (ILM) peeling and gas injection (perfluoropropane[C3F8] or sulfur hexafluoride [SF6]) based on the surgeon’s discretion. Three skilled VR surgeons—two with more than 15 years of surgical experience and one with five—performed the operation. Together, they perform about 100 VR surgeries a year. Additionally, the surgeons have performed more than 30 operations using the 3D surgical system over the course of the last 12 months. All patients underwent thorough clinical evaluations at the baseline and one-month post-operative visits, which included assessing best-corrected visual acuity (BCVA) using the Snellen chart, intraocular pressure measurement (IOP) by Goldmann applanation tonometry, anterior segment and fundus evaluation, and spectral-domain optical coherence tomography (SD-OCT). The pre-operative MH basal diameter (MH-BD) was measured by a single grader using the SD-OCT machine’s built-in calipers.

### Cognitive load assessment

The cognitive workload was measured during surgery with the help of real-time monitoring systems and patient reports. Measurements of HR and oxygen saturation (SPO2) levels were taken with a pulse oximeter and included in the real-time tool. The baseline measurements were assessed two hours before surgery. With the surgeon’s right toe connected to the pulse-oximeter, intraoperative parameters were monitored throughout the operation. The measurements were taken at the conclusion of the ILM peeling stage. Readings were taken 10 min after the operation too. To exclude any bias resulting from baseline parameters, only the first surgery of the day was included in the analysis.

The operating surgeon used the SURG-TLX questionnaire to setlf-report his or her cognitive burden after the surgery [14]. 

### SURG-TLX questionnaire (appendix 1) [14]

“The SURG-TLX assesses six dimensions of workload as follows: [14]

1. Mental demands: How mentally fatiguing was the procedure?
2. Physical demands: How physically fatiguing was the procedure?
3. Temporal demands: How hurried or rushed was the pace of the procedure?
4. Task complexity: How complex was the procedure?
5. Situational stress: How anxious did you feel while performing the procedure?
6. Distractions: How distracting was the operating environment?”

SURG-TLX is calculated using a two-part evaluation. As a first step, 15 pairwise comparisons are made between the dimensions to assess the significance and relevance for every surgery (Appendix 1). This is called the weight of the dimension, and it can be between 0 and 5. In the second portion, the surgeon assigns a score on a Likert scale that ranges from 0 to 20, with 0 indicating the lowest and 20 indicating the highest. This is referred to as the rating of the dimension. Following that, the sum of these two numbers is used to generate the workload score for each dimension. A weighted score of 3 and a rating of 15, for example, equal a workload score of 45. (Scores range from 0 to 100). Combining the scores from the six dimensions yields a score for the total workload (Scores range from 0 to 600).

### Surgical parameter comparison

The total duration of the surgery, duration of the ILM peel, complications, MH closure rate, and final visual acuity (VA) outcomes at the end of one month were compared between the two groups.

The Statistical Package for Social Sciences (SPSS) 23.0 version was used to perform the statistical analysis. For statistical purposes, the Snellen BCVA was converted to Logarithm of the Minimum Angle of Resolution (LogMAR) BCVA. Continuous variables were described as mean and variance from the mean (SD) or median and interquartile range (IQR) if they did not follow a normal distribution. Variables with a normal distribution were analyzed with an unpaired t-test, whereas those not following normal distribution were analyzed with the Mann-Whitney U test. Paired T-test analysis was performed on paired variables. Categorical variables were expressed using percentages and analyzed using the Chi-square test or Fisher exact test as appropriate. A repeated measures ANOVA was used to test differences in the heart rate and SPO2 levels between the baseline, intraoperative, and at the end of the surgery. A *p*-value of 0.05 was considered statistically significant.

## Results

The study involved 50 eyes from 50 patients. Twenty eyes (40%) were operated on using the 3D system, while the remaining 30 eyes (60%) were operated on using the conventional microscope method. The mean age of the study participants was 65.46 (± 8.24) years (Table 1). These consisted of 54% women and 46% men. Patients in the 3D group were significantly older than those in the Conventional microscope group (Mean age − 3D group: 68.45 ± 5.89 years; Conventional microscope group: 63.47 ± 9.17 years; *p* = 0.023) (Table 1). The MH-BD between the two groups did not significantly differ (Mean MH-BD– 3D group: 1050.6 ± 196.69 μm; Conventional microscope group: 1145.77 ± 234.67 μm; *p* = 0.128) (Table 1). The BCVA at baseline was significantly better in the 3D group than in the Conventional microscope group. (Mean LogMAR BCVA– 3D group: 1.01 ± 0.54; Conventional microscope group: 1.35 ± 0.5; *p* = 0.027).

**Table 1:** Comparison of baseline variables between the three-dimensional and conventional microscope surgical groups

Baseline Variables			3D Surgery (*N* = 20)	CM Surgery (*N* = 30)	*P* value
**Age**		Mean ± SD	68.45 ± 5.89	63.47 ± 9.17	0.023*
**Gender**	Males	Number (Percentage)	11 (55)	12 (40)	0.137
	Females		9 (45)	18 (60)	
**MH-BD (µm)**		Mean ± SD	1050.6 ± 196.69	1145.77 ± 234.67	0.128

3D: Three-dimensional; CM Conventional Microscope; SD: Standard deviation; MH-BD: Macular hole basal diameter; *: Significant *p*-value

### Cognitive load assessment: real-time tools

In both groups, mean HR increased significantly intraoperatively and postoperatively when compared to baseline (Table 2). Intraoperative SPO2 was likewise significantly greater than baseline in both groups (Table 3). The postoperative SPO2 values, however, did not significantly differ from the baseline (Table 3). The mean intraoperative HR was significantly higher in the Conventional microscope group than in the 3D group (Mean HR– 3D group: 79.15 ± 4.49; Conventional microscope group: 82.27 ± 4.81; *p* = 0.024) (Table 2). The heart rates (HR) and SPO2 levels of the two groups were similar pre- and post-surgery (Table 2).

**Table 2:** Comparison of heart rate (HR), oxygen saturation (SPO2) levels, and best-corrected visual acuity (BCVA) between the three-dimensional and conventional microscope surgical groups

			3D Surgery (*N* = 20)	*P* value Intragroup compared with baseline	CM Surgery (*N* = 30)	*P* value Intragroup compared with baseline	*P* value between 2 groups
**Heart Rate**	Baseline	Mean ± SD	67.4 ± 5.96	NA	69.4 ± 6.07	NA	0.255
	Intraoperative (ILM Peel)		79.15 ± 4.49	< 0.00001*	82.27 ± 4.81	< 0.00001*	0.024*
	Postoperative		75.45 ± 7.39	0.0005*	72.37 ± 5.06	0.281*	0.086
**SPO2 (%) levels**	Baseline	Mean ± SD	93.6 ± 6.1	NA	91.87 ± 5.1	NA	0.301
	Intraoperative (ILM Peel)		97.8 ± 1.1	0.0067*	97.57 ± 1.22	< 0.00001*	0.487
	Postoperative		94.1 ± 5.12	0.79	93.83 ± 5.53	0.185	0.862
**BCVA**	Pre-operative	Mean ± SD	1.01 ± 0.54	< 0.001*	1.35 ± 0.5	< 0.001*	0.027*
	Post-operative		0.5 ± 0.25	< 0.001*	0.54 ± 0.24	< 0.001*	0.515

3D: Three-dimensional; CM: conventional microscope; SD: Standard deviation; ILM: Internal limiting membrane; *: Significant *p*-value (repeated measures ANOVA test)

**Table 3:** Comparison of the Surgery Task Load Index (SURG-TLX) between the three-dimensional and Conventional micrscope surgical groups

SURG-TLX Parameters		Group 1 (*N* = 30)	Group 2 (*N* = 20)	*P* value
**Mental Demands WS**	Median (IQR)	22.5 (15–40)	15 (10–28.75)	0.153
**Physical Demands WS**		20 (13.75–30)	25 (16.25–33.75)	0.095
**Temporal Demands WS**		15 (10–35)	10 (0–15)	0.004*
**Task Complexity WS**		20 (10–30)	12.5 (6.25–23.75)	0.137
**Situational Stress WS**		25 (15–31.25)	20 (10–25)	0.318
**Distractions WS**		25 (10–30)	20 (10–25)	0.412
**Total WS**	Mean ± SD	137.17 ± 28.82	113 ± 29.39	0.005*

SURG-TLX: Surgery Task Load Index; 3D: Three-dimensional; CM Conventional microscope; WS: Workload Score; IQR: Inter-quartile range; SD: Standard deviation; *: Significant *p*-value

### Cognitive load assessment: SURG-TLX analysis (table 3)

In the Conventional microscope group, the surgeon’s mean total workload score was significantly greater than in the 3D group (Mean total workload score– Conventional microscope group: 137.17 ± 28.82; 3D group: 113 ± 29.39; *p* = 0.005). Additionally, the median workload score of the ‘temporal demands’ dimension was significantly higher in the Conventional microscope group than in the 3D group (Conventional microscope group: 15 [IQR 10–35]; 3D group: 10 [IQR 0–15]; *p* = 0.004). For the other five dimensions of the SURG-TLX questionnaire, there was no significant difference between the two groups’ workload scores. Using SURG-TLX Analysis, there was no difference amongst surgeons.

### Duration of surgery (table 4)

**Table 4:** Comparison of duration and number of intraoperative capillary bleeds between the three-dimensional and Conventional microscope surgical groups

Parameter		3D Surgery (*N* = 20)	CM Surgery (*N* = 30)	*P* value
Total Surgical Duration (seconds)	Mean ± SD	3723 ± 573.34	3552.41 ± 535.31	0.299
Duration of ILM peel (seconds)	Mean ± SD	442.14 ± 31.09	446.18 ± 37.69	0.682
Number of Hemorrhages	Median (IQR)	1 (0–2)	1 (0–2)	0.75

3D: Three-dimensional; CM; Conventional Microscope SD: Standard deviation; IQR: Inter-quartile range; ILM: Internal limiting membrane

Both the mean total surgical time and the mean ILM peel time were comparable in the groups using the 3D and conventional microscopes (Total surgical duration − 3D group: 3723 ± 573.34 s; Conventional microscope group: 3552.41 ± 535.31 s; p: 0.299; Mean ILM peel time– 3D group: 442.14 ± 31.09 s; Conventional microscope group: 446.18 ± 37.69 s; p: 0.682). (Table 4)

### Complications and surgical outcomes

There were no complications during or after surgery in either group. The median number of capillary bleeds did not differ between the 3D and conventional microscopy groups (Median capillary bleeds– Conventional microscope group: 1 [IQR 0–2]; 3D group: 1 [IQR 0–2]; *p* = 0.75) (Table 5). Two cases in the 3D group (2/20; 10%) and 3 cases in the Conventional microscope group (3/30; 10%) had non-closure of MH at the one-month post-operative visit. At the one-month post-operative visit, MH did not close in two cases in the 3D group (2/20; 10%) and three cases in the conventional microscopy group (3/30; 10%).

**Table 5:** Comparison of duration and number of intraoperative capillary bleeds between the three-dimensional and Conventional microscope surgical groups

Parameter		3D Surgery (*N* = 20)	CM Surgery (*N* = 30)	*P* value
Total Surgical Duration (seconds)	Mean ± SD	3723 ± 573.34	3552.41 ± 535.31	0.299
Duration of ILM peel (seconds)	Mean ± SD	442.14 ± 31.09	446.18 ± 37.69	0.682
Number of Hemorrhages	Median (IQR)	1 (0–2)	1 (0–2)	0.75

3D: Three-dimensional; CM; Conventional Microscope SD: Standard deviation; IQR: Inter-quartile range; ILM: Internal limiting membrane

### Visual acuity outcomes (table 2)

The mean LogMAR BCVA at one month showed significant improvement in both groups compared to the baseline (*p* < 0.001 in both groups). The final BCVA, however, did not differ between the 3D and Conventional microscope groups.

## Discussion

In this study comparing the 3D and Conventional microscope visualization systems for MH surgery, we found that the 3D technology reduces the surgeon’s cognitive workload significantly. Additionally, our study demonstrates that the type of viewing system has no bearing on the overall surgical time, ILM peel time, surgical complications, visual acuity results, or MH closure rates.

3D stereoscopic surgical systems were introduced in the 1990s for minimally invasive surgeries such as laparoscopic surgeries, small-incision abdominal surgeries, and robotic surgeries [16]. After researching a Three-dimensional On-screen Microsurgical System (TOMS), Franken RJ et al. (1995) proposed using this cutting-edge technology for ocular surgeries [17]. The 3D system has subsequently been used for numerous vitreoretinal and anterior segment surgeries during the past ten years [8, 9]. Ophthalmic surgeons are becoming more open to this 3D technology because it has many advantages over the traditional conventional microscope system, such as better ergonomics, improved depth perception, and lower illumination levels that make patients more comfortable and reduce retinal phototoxicity. A heads-up display system also lets you train and teach more than one observer at the same time. Residents and other trainees should find this very advantageous since they will be able to learn from the same viewing system that provides the same depth of perception as the primary surgeon. A further benefit of this system, particularly in vitreoretinal surgeries, is the use of multiple filters.

The 3D system offers superior ergonomics, illumination settings, depth of field, display filters, and trainee experience, according to Agranat JS et al’s [18] review of 272 VR surgeries. The indications for surgery in their series included macular surgeries such as MH, epiretinal membrane (ERM) and vitreomacular traction (VMT), proliferative diabetic retinopathy (PDR) with/without tractional retinal detachments (TRD), vitreous hemorrhages (VH), rhegmatogenous retinal detachments (RRD), silicone oil removal, and so on [18]. Talcott KE et al. found that for MH procedures, even though the total surgical length was comparable between the 3D heads-up display (3D HUD) surgical platform and a standard operating microscope (SOM), the macular peel time with the 3D HUD was much longer than with the SOM [7]. While we did not observe any significant differences in the total surgical duration between the two groups, we did find that the ILM peel time was comparable across the 3D and the Conventional microscopy groups. Comparable overall surgical time and total ILM peel time were also demonstrated by Kumar A et al. [9] in a similar investigation comparing the 3D and Conventional microscopy systems. Furthermore, in their study, type 1 closure was observed in 92% of the 3D group eyes and 88% of the conventional microscope group eyes. [9] In our series, we had similar closure rates of 90% in both arms. The final visual acuity did not differ between our two groups, which was consistent with the findings of Kumar A et al. [9] Furthermore, none of our eyes in either arm experienced any intraoperative or postoperative complications.

Despite having a potentially infinite capacity, the human brain can only handle a finite quantity of information at one time [19]. This is referred to as the “working memory.” [19] Cognitive load is a measurement of how much of this working memory is being used. The excessive workload in the form of difficult surgeries, intraoperative complications, anxiety related to the operating room atmosphere, and surgical flow disruption owing to equipment or viewing system difficulties can all be detrimental to the surgeon. The surgeon’s cognitive workload can be estimated in real-time using tools like HR, SPO2 levels, and EEG as well as through self-report methods like SURG-TLX and SWAT [13]. Romano MR et al. analyzed the surgeon’s satisfaction as well as the safety and efficacy of 50 eyes undergoing VR operations for diverse indications [15]. Based on a questionnaire that examined seven factors—comfort, visibility, image quality, depth perception, simplicity of use, maneuverability, and teaching—they evaluated the surgeon’s satisfaction [15]. Although they indicated a higher level of satisfaction with the 3D system [15], the lack of an authentic assessment method made the results debatable. To address this issue, we used a validated questionnaire called SURG-TLX to assess the surgeon’s cognition, along with additional physiological real-time tools including HR and SPO2 levels.

Performing VR surgery is a difficult task that may be accompanied by varying amounts of stress. This aspect better explains why the HR levels were much higher during surgery and after surgery in both groups of our study. Additionally, we observed that the intraoperative HR during ILM peeling was significantly higher in the conventional microscope group compared to the 3D group. As a result, we may infer that the surgeon felt more comfortable and at ease during the operation while using the 3D system. This may be because of a variety of causes, such as improved ease and ergonomics, enhanced visualization of the ILM due to greater depth perception, the usage of filters, reduced illumination, as well as possibly better anatomical visualization. Even the subjective workload measured by the SURG-TLX questionnaire showed a much lower total workload score when the surgeon used the 3D operating system instead of the Conventional microscope system. The “temporal demand” score, which measures “the amount of time pressure involved in completing the task”, [15] was significantly lower with the 3D system among the six dimensions assessed by this questionnaire. According to the results of the self-assessment tool, the surgeon’s cognitive effort was significantly decreased with the 3D operating system, whereas he felt rushed for time doing the MH procedure with the Conventional microscopy equipment.

In both the 3D and the conventional microscope systems, the surgeon’s SPO2 levels increased during the procedure and then decreased to preoperative levels after the procedure. Humans, as demonstrated by V. Perciavalle et al., [20] engage in deep breathing on their own accord as a means of lifting their moods and calming their nerves. This can result in an increase in the mean SPO2 levels [21]. So, higher intraoperative SPO2 levels in our study could be due to the surgeon’s reflex of deep breathing to reduce his stress.

Despite the 3D surgical systems offering multiple advantages, they do come with their unique challenges. Firstly, the elevated cost of 3D surgical instruments poses a significant financial barrier, limiting their availability primarily to a select few large, specialized hospitals. Also, presently, there exists an opportunity for enhancement in the management of certain peripheral retinal lesions during 3D surgery, particularly in addressing issues such as distortion and a deficiency in stereoscopic vision during peripheral vitrectomy. The collaborative efforts of the surgical team are challenged by the need for substantial head tilting by the assistant when dealing with peripheral lesions. Finally, the optimal functionality of the 3D surgical system requires meticulous alignment with the microscope system to mitigate potential malfunctions during surgery. Adjustments necessitating a microscope restart may contribute to prolonged surgical times, underscoring the importance of seamless integration between these components.

The major limitations of this study are the small sample size, the fact that the ergonomics of the surgeons were not properly evaluated, either subjectively or objectively, and the fact that there was no evaluation of the patients’ comfort and satisfaction levels during surgery. This study’s strengths, however, lie in its prospective design and its evaluation of the newer 3D surgical viewing method in comparison to the conventional microscopic technique. Furthermore, this is a one-of-a-kind study to analyze and compare the surgeon’s physiological parameters and workload analyses across the two viewing systems utilizing the unique SURG-TLX questionnaire.

To summarize, when compared to the traditional Conventional microscope viewing system, performing MH surgery using the modern 3D visualization systems (Artevo) improves the surgeon’s cognitive workload without compromising total surgical duration, ILM peel time, visual acuity outcomes, and MH closure rates. Additional research on the cognitive workload of surgeons, as well as the ergonomics and patient satisfaction levels with this newer 3D technology across the entire spectrum of VR surgeries, is warranted.

### Electronic supplementary material

Below is the link to the electronic supplementary material.

Supplementary Material 1

## Data Availability

The datasets used and/or analyzed during the current study available from the corresponding author on reasonable request.
