# Supplementary material for: Comparison of cognitive workload and surgical outcomes between a three-dimensional and conventional microscope macular hole surgery
Source: BMC Ophthalmol. 2024 Mar 1;24:95. doi: 10.1186/s12886-024-03361-5 (PMC10908162; doi:10.1186/s12886-024-03361-5)
Supplement: Supplementary file 1 — Supplementary Material 1 [file 12886_2024_3361_MOESM1_ESM.pdf]

Appendix 1: Surgery Task Load Index (SURG-TLX) questionnaire

Weighted rating

There are six rating scales which are meant for evaluating your experience during the procedure. Please evaluate the procedure by marking "X" on each of the six scales at the point which best fit your experience. The scale ranges from "Low" on the left to “High" on the right. Please read the descriptions carefully.

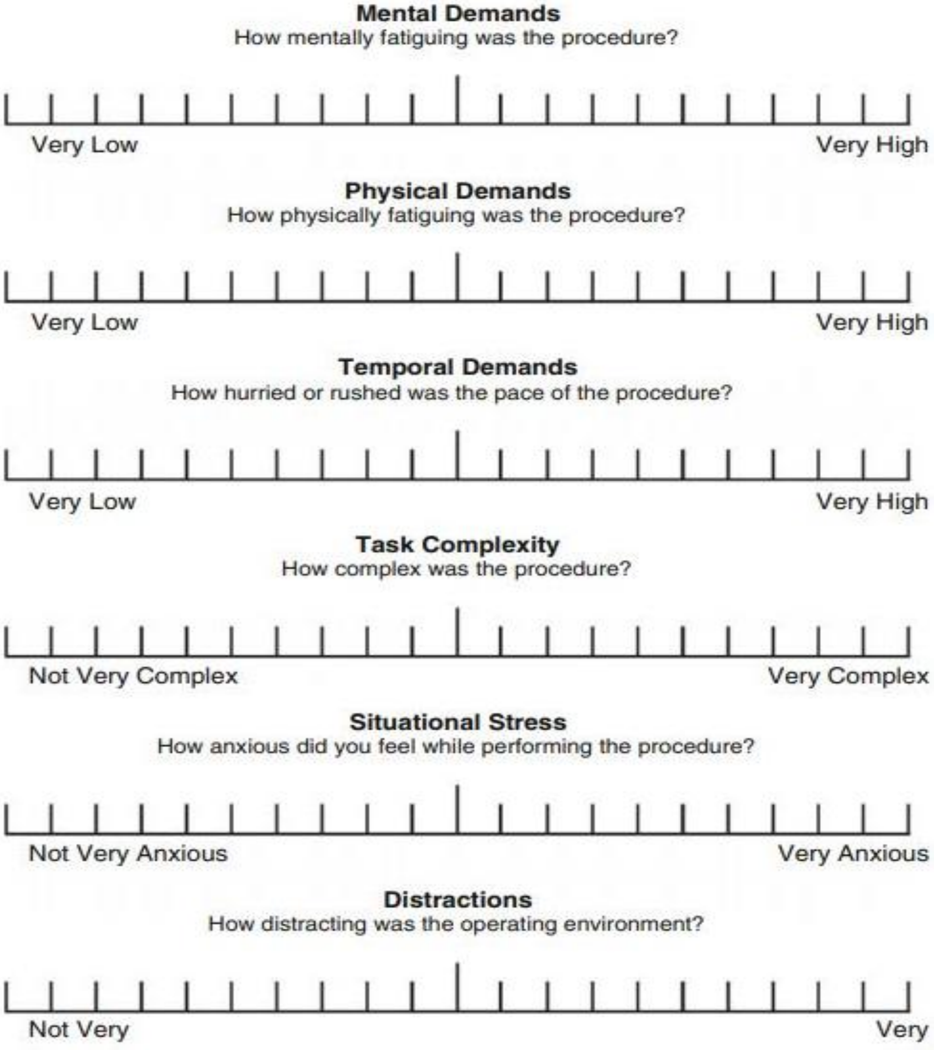

Pairwise comparisons

Following are a set of titles listed into boxes within a grid. From these boxes. you will choose which title you deem more applicable to your experience of workload in the procedure. Circle the title that you deem fitting of your experience. Please consider your choices carefully and make them consistent with how you used the rating scales. We are not looking for a right or wrong answer. We are only interested in your opinion.

Task Complexity<br>Or<br>Mental Demand	Distractions<br>Or<br>Situational Stress	Task Complexity<br>Or<br>Distractions
Task Complexity<br>Or<br>Temporal demand	Mental demand<br>Or<br>Situational Stress	Physical Demand<br>Or<br>Distractions
Mental demand<br>Or<br>Physical demand	Situational Stress<br>Or<br>Physical demand	Situational Stress<br>Or<br>Task Complexity
Temporal demand<br>Or<br>Mental demand	Distractions<br>Or<br>Mental demand	Temporal demand<br>Or<br>Distractions
Physical demand<br>Or<br>Temporal demand	Physical demand<br>Or<br>Task Complexity	Temporal demand<br>Or<br>Situational Stress
